# 

**Published:** 1991-06

**Authors:** 


Bristol Medico-Chirurgical Society

### WEST OF ENGLAND MEDICAL JOURNAL

**JUNE 1991** **Volume 106 (ii)**

Copyright © West of England Medical Journal Ltd 1991.

**Contents**

30 **The Changing Face of Bristol Medicine**
A symposium organised by the Bristol Medico-Chirurgical Society

33 **Four Hunterian Lectures and Bristol – 1990**
N.M.P. Clarke, R. Miller, S. Nicholson, D.J.A. Scott, J.R. Farndon.

37 **Screening for Disease**
Peter Skrabanek

40 **Towards Better Discharge Summaries**
M.H. King, S.G. Barber

42 **Perforation of a Peptic Ulcer in an Hiatus Hernia**
A.J. Hamer, E. Sheffield

43 **Non-Immune Hydrops**
John Smoleniec and David James

44 **Recurrent Radial Head Dislocation**
Barbara Michelman

45 **Book Reviews**
Second opinions in internal Medicine – Sandler, Fry, Fabb and Godfrey. Lecture Notes on the Physics of Radiology – Armstong. Essentials of Dermatology – Burton

46 **Surgical Club of S.W. England**
Meeting at Weston-super-Mare, October 1990

49 **S.W. Vascular Surgeons**
Meeting aqt Swansea, February 1990

51 **S.W. Orthopaedic Club**
Meeting at Exeter, April 1991

53 **S.W. Radiologists Association**
Meeting at Gloucester in October 1990

***What I know remains unknown unless by me to others shown.***
**Lucilius (180–102 BC)**

